# Interleukin-33: A Double-Edged Sword in Sepsis

**DOI:** 10.31480/2330-4871/188

**Published:** 2024-09-16

**Authors:** Shuai Liu, Yinyan Yue, Qiuge Wu, Li-Ming Zhang

**Affiliations:** 1 Department of Pulmonary and Critical Care Medicine, The First Affiliated Hospital of Zhengzhou University, Zhengzhou, Henan, China; 2 Department of Anesthesiology & Perioperative Medicine, University of Pittsburgh School of Medicine and University of Pittsburgh Medical Center, Pittsburgh, Pennsylvania, USA

**Keywords:** Interleukin-33 (IL-33), Pro-inflammatory, Anti-inflammatory, Sepsis

## Abstract

Despite some advances that have been made in the clinical management of sepsis, its morbidity and mortality rates remain high. The pathogenesis of systemic inflammation and organ damage leading to septic mortality is not fully understood. Interleukin-33 (IL-33), a new member of the IL-1 superfamily, can be a traditional cytokine and a nuclear factor regulating gene transcription. Due to its biological characteristics, IL-33 can act as a double-edged sword in sepsis: It not only contributes to clear bacteria and improves survival but also causes inflammation, immunosuppression, and organ damage via modulating immune response. In this review, we will summarize updated information on the dual functions of IL-33 in the host immune response to sepsis.

## Introduction

In 2005, Interleukin-33 (IL-33) was identified as a new member of the interleukin (IL)-1 cytokine family [1]. IL-33 is constitutively and abundantly expressed in bronchial and intestinal epithelial cells, endothelial cells, and fibroblasts in human [2,3]. Under resting conditions, IL-33 is localized in the nucleus, as mediated by the presence of an N-terminal domain nuclear localization sequence. Since IL-33 can function both as an intracellular nuclear factor and a conventional cytokine, which makes it a particular characteristic role in transcriptional regulation and activation of the ST2 receptor complex signal. IL-33 has been proposed to be another alarmin with dual functions, similar to HMGB1 and IL-1α. Accumulated experimental evidence indicates that IL-33 is involved in both Th1- and Th2-cells mediated immunity responses and has both pro-inflammatory and anti-inflammatory effects simultaneously [4].

Sepsis remains a leading cause of death in the Intensive Care Unit (ICU) [5]. There is increasing evidence that suggests that IL-33 is involved in the initiation and progression of inflammatory diseases, such as sepsis [6,7]. However, controversy remains with its definitive role in sepsis. In this review, we provide recent advances in IL-33-mediated immune regulation and its effect on sepsis.

## Molecular Features of IL-33

**Figure 1:**
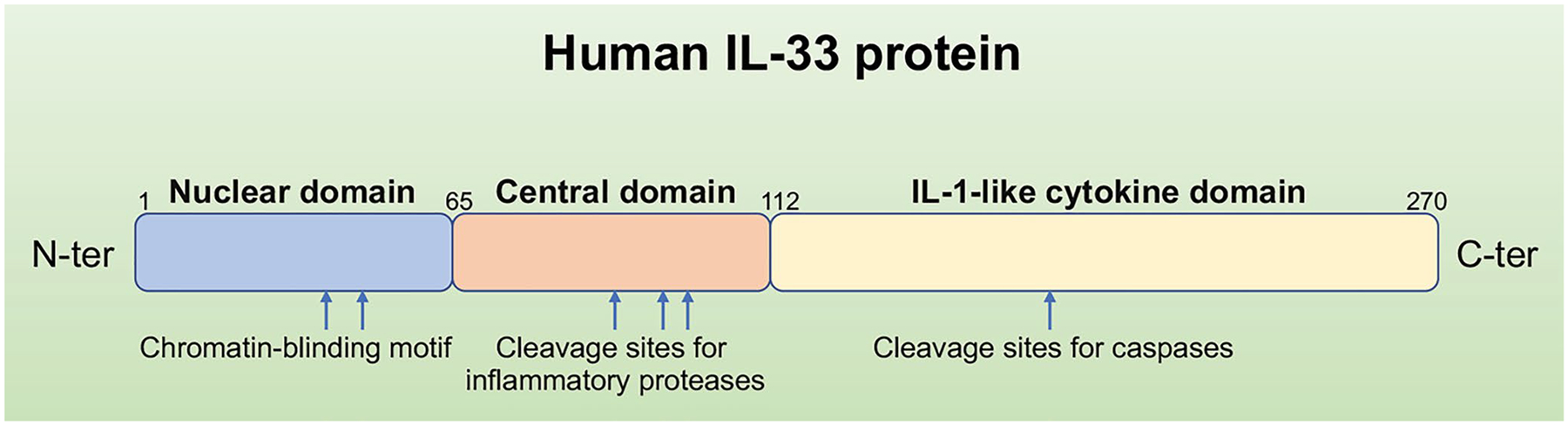
Primary structure of the human IL-33 protein. The full-length IL-33 is a protein with 270 amino acids. It has the N-terminal nuclear structural domain and the C-terminal IL-1-like cytokine structural domain, the cleavage sites for proteases involved in IL-33 activation (inflammatory proteases) or inactivation (caspases) are shown above.

The location of IL33 gene has been mapped to chromosome 9p24.1 in humans, while its mouse homolog is found on the syntenic chromosome 19qC1 region [1]. The IL-33 protein is composed of three functional domains: Nuclear domain, central domain, and IL-1-like cytokine domain [8]. Under basal conditions, the N-terminus of IL-33 contains an evolutionarily conserved homeodomain-like helix-turn-helix (HTH) DNA binding domain, which is necessary and sufficient for nuclear targeting and is involved in the repression of transcription that has been ascribed to IL-33 [9]. The central domain of IL-33 is sensitive to neutrophil- and mast cell-derived proteases due to the protease cleavage sites in it [10,11]. As to the C-terminal IL-1-like cytokine domain of IL-33, its binding interface structure plays a critical role in interacting with all three Ig-like domains of the ST2 ectodomain [12], and mediates the cytokine activities [13] (Figure 1). Consistent with the features in structure, thus, IL-33 might have dual functions involved in immune regulation either as a traditional cytokine through activation of the ST2 receptor complex or as an intracellular nuclear factor with transcriptional regulatory properties, similar functions have previously been known for IL-1a and HMBG1 [14].

## Expression of IL-33

During homeostasis, constitutive and abundant expression of IL-33 mRNA and protein is found in the airway, bronchial epithelial cells, endothelial cells of high endothelial venules, fibroblasts, and platelets in human [1,2,15,16]. In contrast, in rodents, constitutive expression of IL-33 is not detected in endothelial cells under basal conditions, however, its expression can be induced in the microenvironment of chronic inflammation, revealing the species-specific differences between human and mouse [3]. Pichery, et al. found that endogenous IL-33 is highly expressed in mouse epithelial barrier tissues (including stratified squamous epithelia from the vagina and skin, cuboidal epithelium from the lung, stomach, and salivary gland), lymphoid organs, brain, embryos during homeostasis [3]. Under the condition of inflammation or tissue stress, the constitutive expression of IL-33 at a high level in tissues can be further upregulated, such as in the lung during papain-induced allergic airway inflammation, and in the liver during LPS-induced endotoxin shock [3].

## Processing and Release of IL-33

**Figure 2:**
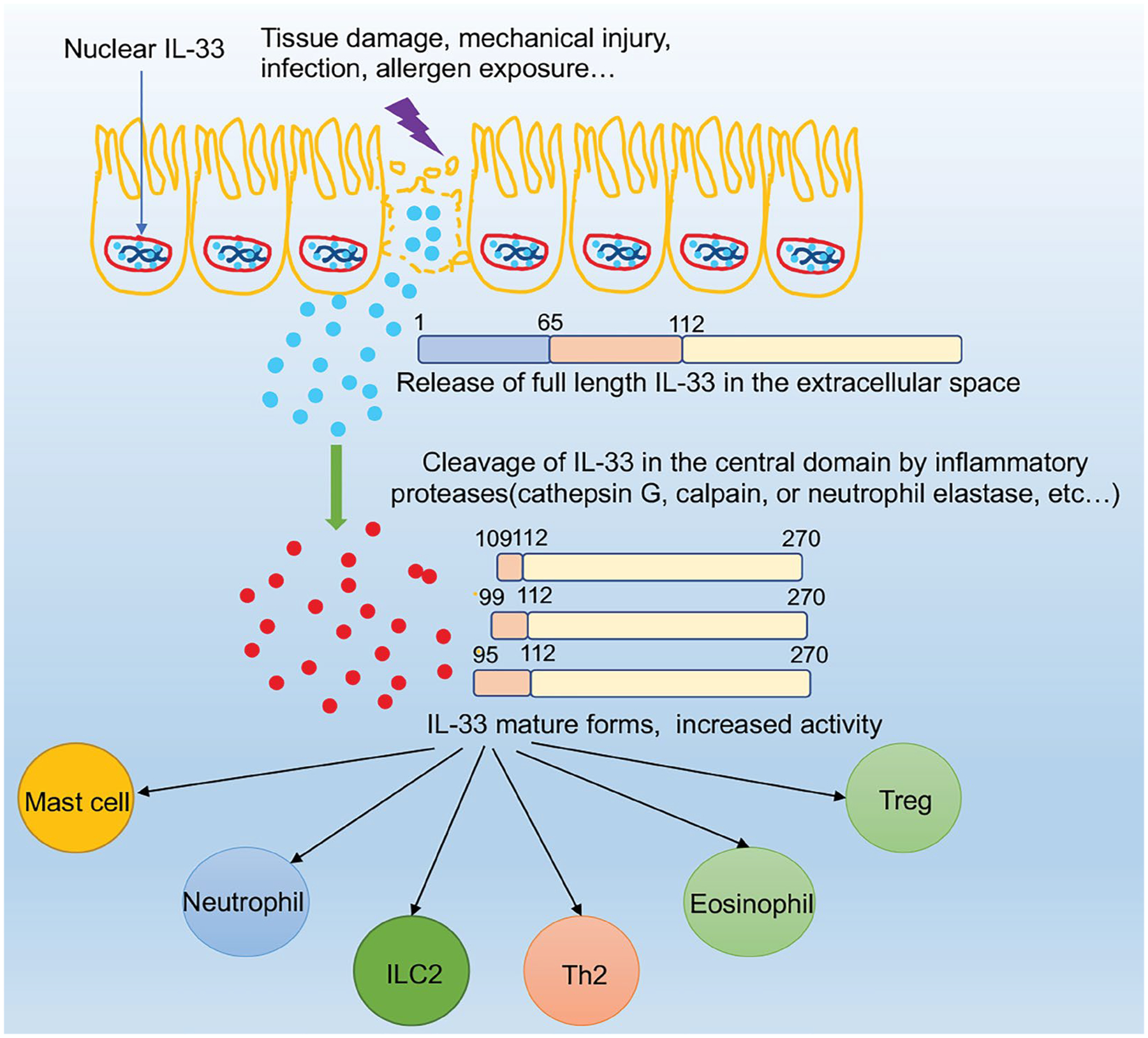
The mechanisms of IL-33 extracellular release. After epithelial cell exposure to tissue damage, mechanical injury, infection, allergen exposure, etc. IL-33 will be quickly released from the nucleus and active the ST2+ cells such as ILC2s. In this process, IL-33 can be cleaved into shorter mature forms by inflammatory proteases such as neutrophils cathepsin G.

While in nuclear, IL-33 has a crucial role in chromatin compaction and repression of gene expression [17]. In addition to repressing gene expression, IL-33 nuclear location was demonstrated to have an ability to potently regulate the extracellular function of IL-33; in the absence of nuclear localization sequestration, IL-33 is sustainably released into the extracellular from the nucleus, which ultimately causes non-resolving lethal inflammation, suggesting that the sub-cellular localization of IL-33 is very important for controlling its extracellular release and maintaining immune homeostasis [18]. Furthermore, because the release of IL-33 is independent of caspase-1, caspase-8, or calpain [19], the nuclear localization of IL-33 becomes even more important: after release, the full-length form of IL-33 is active and may contribute to the pathogenesis of inflammatory response in many diseases. The activity of the full-length form of IL-33 is abolished when processed by caspase-1, caspase-3, or caspase-7 [20,21]. Once the full-length form of IL-33 (30 kDa) is released into the extracellular space, it is cleaved by proteases, such as cathepsin G, calpain, or neutrophil elastase, then leads to the production of C-terminal cleavage products containing the entire IL-1-like domain, finally becomes to a stronger pro-inflammatory form, mature IL-33 (18 kDa) [11] (Figure 2).

Pichery, et al. found that the IL-33 protein was only localized in the cell nucleus rather than in the cytoplasmic of producing cells [3]. However, Kakkar and her colleagues demonstrated that IL-33 localized simultaneously to nuclear euchromatin and cytoplasmic vesicles, newly synthesized IL-33 molecules are initially imported into the nucleus for euchromatin association, from where they gradually efflux into the cytoplasmic space to be packaged into secretory vesicles during biomechanical overload by living cells, which was dependent on nuclear pore complex function [22].

After being released into the extracellular space, the oxidation of IL-33 occurs rapidly by the formation of two disulfide bridges, which leads to the biological activity of IL-33 at its receptor ST2 being terminated [23]. Such a mechanism would limit IL-33 in time and space, restricting its activity to the ST2-dependent immunological responses. This suggests that the activity of IL-33 in mediating inflammatory response is also regulated by post-translational modification.

## IL-33/ST2 Signaling

Once released, IL-33 can induce signaling pathways through binding to its heterodimeric receptor complex which is composed of ST2 and IL-1R accessory protein (IL-1RAP) [24]. ST2, the receptor of IL-33, is a member of the IL-1 receptor (IL-1R) family. The two major protein isoforms encoded by the ST2 gene include a transmembrane full-length form (ST2 or ST2L) and a soluble, secreted form (sST2) [6,25]. Membrane-bound ST2 is thought to be a functional component for IL-33 signaling [6,25], whereas sST2 acts as a decoy receptor for blocking IL-33 signaling due to a lack of transmembrane and intracellular domains [26].

Upon activation, IL-33/ST2/IL-1RAP leads to the recruitment of myeloid differentiation primary response protein 88 (MyD88) [27], interleukin receptor-associated kinase 4 (IRAK4), followed by interaction between IRAK1, IRAK2, and/or IRAK3, together forms the complex called myddosome. This myddosome then interacts with tumor necrosis factor receptor-associated factor 6 (TRAF6) [28,29], leading to the activation of the transcription factors, including nuclear factor-kappaB (NF-κB) and MAP kinases (ERK, p38 and JNK) [1,6,24].

**Figure 3:**
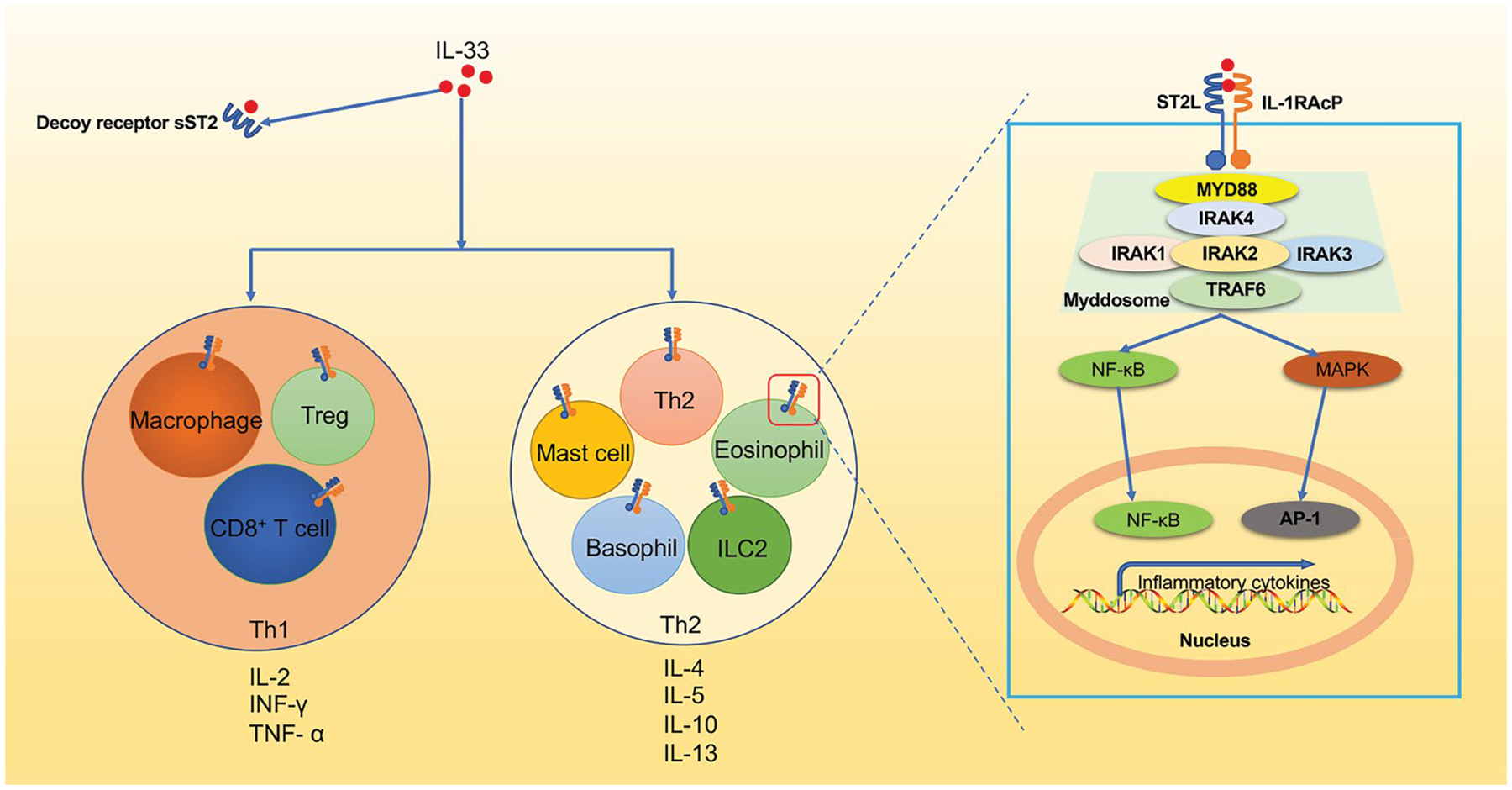
The IL-33-ST2 signaling pathway. IL-33 activates ST2+ Treg, CD8+ T cells, and macrophages to produce TNF-α and IFN-γ to mediate type I immune responses. Additionally, ST2+ mast cells, basophils, eosinophils, ILC2s, and Th2 cells are also activated by IL-33 to produce type 2 cytokines (IL-4, IL-5, IL-9, IL-13) promoting the classical type 2 immune response. At the molecular level, IL-33 forms a complex with ST2 and IL-1 receptor accessory protein (IL-1RAcP). Subsequently, myeloid differentiation primary response protein 88 (MYD88), IRAK4 is then recruited to MyD88, followed by interaction between IRAK1, IRAK2, and/or IRAK3 to form a complex known as the myddosome. This myddosome then interacts with TRAF6 and further activates the transcription factors NF-κB or MAPK, which lead to NF-κB and AP-1 pathways respectively.

Subsequently, IL-33 induces the production of various pro- or anti-inflammatory mediators such as IL-6, TNF-α, IL-1β, IL-5, and IL-13 in Th2 cells, eosinophils, basophils, and mast cells, et al. [27,30–32]. Furthermore, IL-33 was also involved in driving both Th1 and Th2 immune responses through its activity on a variety of immune cells under specific microenvironments, which eventually results in a complex range of biological functions in different diseases [6,7] (Figure 3).

## The Role of IL-33 in Sepsis

Clinical data have shown elevated serum levels of IL-33 or sST2 in adult patients with sepsis, whereas more serum sST2 levels were present in the non-survivors compared to the survivors [33–36]. There are significantly higher levels of IL-33 or sST2 in blood in the early phase of childhood sepsis [37,38]. Ziehe’s results showed that the increase in IL-33 concentration was positively correlated with the expression of aquaporin-3 (AQP3), accompanied by a shortened survival period in patients with sepsis [39]. Even though some scholars have concluded that sST2 level is a valuable marker in assessing the severity and mortality of sepsis [40–42], even identifying the etiology of shock between septic shock and cardiogenic shock in the early phases [43]. Additionally, elevated levels of ST2 are associated with a higher risk of hyperglycemia in mechanically ventilated septic patients [44]. However, further research is needed to elucidate whether IL-33 can be used as a biomarker to diagnose, assess the severity, and predict the outcome of sepsis.

Furthermore, some animal experimental studies showed that IL-33 plays a protective role against sepsis. In a sepsis model induced by cecal ligation and puncture (CLP), the administration of exogenous IL-33 enhances bacterial clearance, leading to improved survival of septic mice; at 24 h after CLP, IL-33 attenuates the system inflammation, represented by the decreased levels of IL-6, IL-10, TNF-α, and IFN-γ in serum, as well as the severity of organ injury, which was likely due to IL-33 prevented apoptosis of T lymphocytes and improved bacterial clearance [45]. Recombinant IL-33 protects against LPS-induced endotoxemia and liver injury by increasing the number of liver-infiltrating ST2+ Tregs and promoting the early resolution of excessive inflammation [36]. Lai, et al. found that IL-33/ST2 signaling increases the expression of GRK2 and decreases the level of CXCR4, mediating ILC2 expansion in the lungs and subsequently secretion of IL-9, which plays an important role in protecting lung endothelial cells from pyroptosis in sepsis mice [46,47]. It is also reported that IL-33 treatment suppressed pro-inflammatory cytokines production and improved the survival rate in sepsis mice by suppressing the IL-17 pathway through activating the suppressor of cytokine signaling (SOCS)-3 [34]. Moreover, exogenous IL-33 treatment can increase the neutrophil influx into the peritoneal cavity, which contributes to enhancing bacterial clearance in the site of infection and reducing mortality in mice with sepsis from CLP [48]. Interestingly, the recruitment of neutrophils influx from the circulation to the site of infection was mediated by the CXCR2, a chemokine receptor on neutrophil, which was down-regulated via activation of Toll-like receptors (TLRs) in circulating neutrophils during severe sepsis [49]. But IL-33 can reverse the down-regulation of CXCR2 expression and promote neutrophil recruitment by repressing the expression of G protein-coupled receptor kinase-2 (GRK2) [48], a serine-threonine protein kinase that induces internalization of chemokine receptors [50,51]. Similarly, increased neutrophil influx into the focus of infection caused by IL-33 contributes to reducing bacterial loads was also observed when sepsis mice infected with gram-negative bacteria or acute *Staphylococcus aureus* [52,53]. Most recently, Gong, et al. demonstrated that Sphingosine-1-phosphate receptor 2 (S1PR2) deficiency modulated the type 2 immune response by promoting the IL-33 increase in macrophage, accompanied by reduced lung injury in post-septic mice [54]. Taken together, an early phase of neutrophil-mediated bacterial clearance seems to be a powerful strategy for IL-33 to benefit bacterial sepsis in the early phases. However, in the late phase of uncontrolled sepsis, the downside of the sustainable enhanced neutrophil recruitment mediated by IL-33 may cause secondary remote organ damage in the late phase of sepsis. Thus, precise modulation in different stages of disease progression is very critical.

Contrary to the view mentioned above, some scholars have found that IL-33 plays a role in promoting the progress of sepsis. IL-33 was found to drive systemic inflammation, ILC2 IL-5 expression as well as neutrophil and monocyte infiltration and cytokine production in the lungs, which identifies IL-33 as a driver of systemic and pulmonary inflammation during intra-abdominal sepsis [55]. IL-33 disturbs medullary thymic epithelial cell (mTEC)/cortical TEC (cTEC) compartment, which results in immunosuppression by inducing thymic involution-associated naive T cell aging and impairs host control of severe infection in mouse disease models of schistosomiasis or sepsis [56]. IL-33-deficient mice were resistant to endotoxin shock *in vivo* and showed a substantially diminished LPS-induced systemic inflammatory response (IL-6, IL-1α, and IL-1β) at 9 and/or 48 h after LPS stimulation *in vitro* [57]. Recombinant IL-33 further enhanced the LPS-induced inflammatory cytokine releasing (IL-6, TNF-α, and IL-1β) in mouse macrophages in an ST2-dependent manner [58,59]. The reason why IL-33 was able to enhance macrophages response to LPS is being linked to IL-33 treatment triggers an increase in the expression of the LPS receptor components (MD2/CD14 and TLR-4) at the plasma membrane, the level of the soluble form of CD14 (sCD14) and the MyD88 adaptor molecule [58]. Apart from the enhanced expression of pro-inflammatory factors (e.g., iNOS, IL-6, and TNF-α) induced by LPS in bone marrow-derived macrophages (BMDMs), IL-33 was showed directly activate macrophage the expression of MHC class I, MHC class II, CD80/CD86, as well as inducible NO synthase (iNOS) in a dose-dependent manner [60]. Moreover, Nascimento’s results showed that endogenous IL-33, released during sepsis, induces the switch of macrophages toward M2 polarization, which has an essential function in the expansion of the Treg cell population via the production of IL-10 [33]; another study found that exogenous IL-33 resulted in the expansion of TIGIT+ Tregs depending on the STAT6 and M2 macrophages, contributing to the development of long-term sepsis-induced immunosuppression [61]. In addition, IL-33 facilitates the level of macrophage pyroptosis by activating the NF-κB/p38 MAPK signaling pathway and increases the mortality of mice with sepsis [62].

Our recent study indicates that in a two-hit model of CLP followed by moderate mechanical ventilation (MTV) or low tidal volume (LTV), the extent of inflammation and injury correlated with intra-pulmonary IL-33 levels and deletion of IL-33 or ST2 attenuated lung injury in the two-hit model [63]. Consistently, our *in vitro* experiments also found that cyclic stretch was able to induce IL-33 production through the HMGB1/TLR-4 signaling pathway in murine respiratory epithelial cells [64]. Furthermore, in our previous study, we have identified that WNT1-inducible signaling pathway protein 1 (WISP1) plays an important role in ventilator-induced lung injury (VILI) and CLP-induced ALI [65–67]. Interestingly, in our most recent study, we found that IL-33 can mediate the expression of WISP1 by activating AKT- and ERK-GSK-β-catenin signaling pathways in macrophage via the interaction of β-catenin/TCF/CBP/p300 in the nucleus, which finally contributes to the occurrence and development of lung injury in the two-hit lung injury model [68]. These findings place IL-33 as a proximal driver of lung injury induced by mechanical ventilation in intra-abdominal sepsis. Understanding the intrinsic link between IL-33, mechanical ventilation, and sepsis is fundamentally important for the development of rational strategies for the treatment of different stages of sepsis and the prevention of its associated complications.

## Conclusion

**Figure 4:**
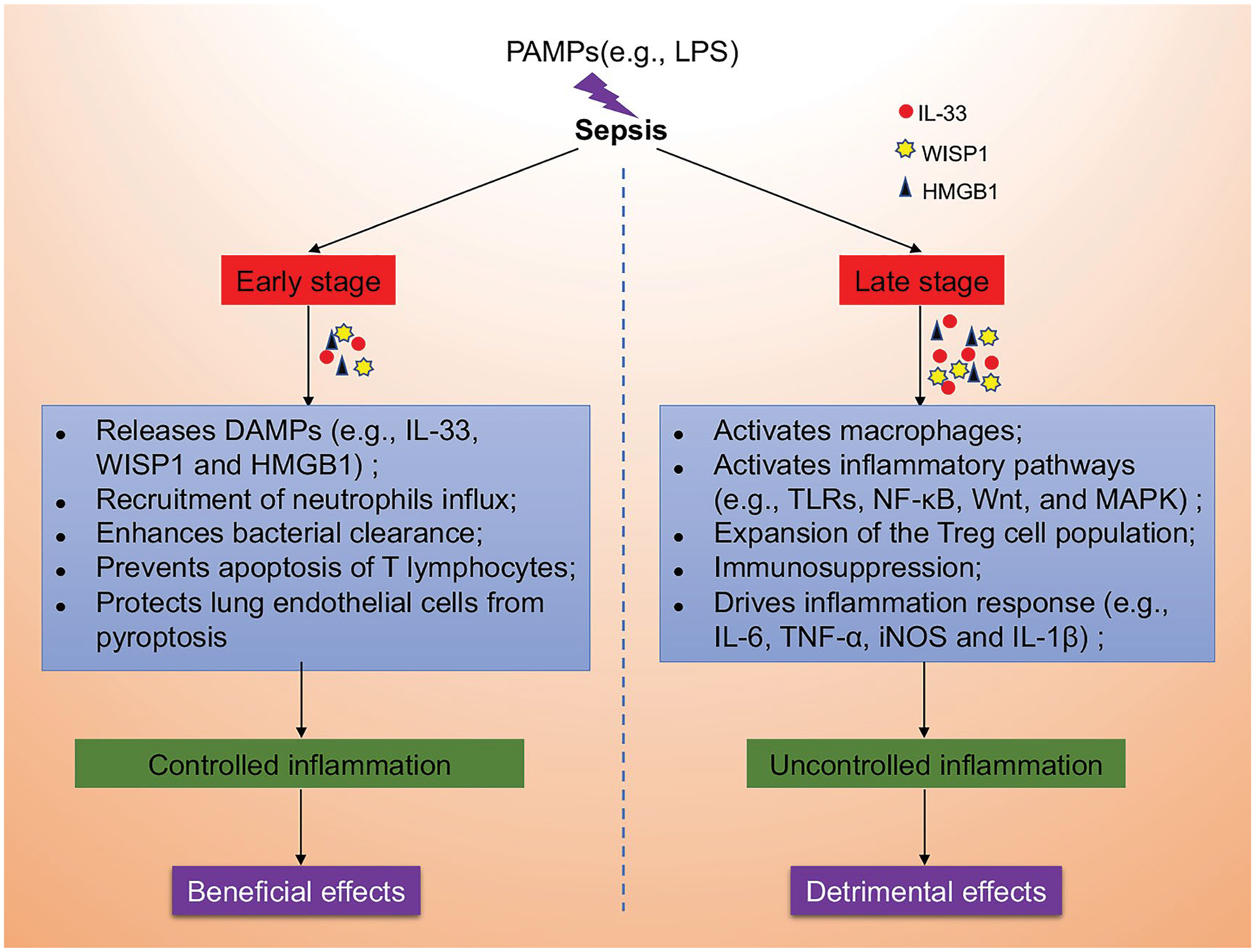
The dual-function of IL-33 in sepsis. We speculate that pathogen-associated molecular patterns (PAMPs, e.g., LPS) induce the release of damage-associated molecular patterns (DAMPs, e.g., IL-33, WISP1 and HMGB1) in the early stage of sepsis, which plays a beneficial role in by recruiting neutrophils influx, enhancing bacterial clearance, preventing apoptosis of T lymphocytes and protecting lung endothelial cells from pyroptosis in controlling inflammation. However, with the development of sepsis towards late stage, the sustainable and increased release of IL-33 may exert detrimental effects through enhanced and uncontrolled inflammation by activating macrophages, activating inflammatory pathways (e.g., TLRs, NF-κB, Wnt, and MAPK), expanding of the Treg cell population, inducing immunosuppression and driving inflammation response (e.g., IL-6, TNF-α, iNOS and IL-1β).

Taken together, these findings suggest that IL-33 plays an essential dual-functional role in the progression of sepsis, the role of IL-33 in sepsis appears to be time and concentration-dependent (Figure 4). However, the exact function and potential mechanism of the IL-33 signaling pathway in sepsis remains to be elucidated. Further investigation of the role of IL-33 in sepsis may lead to the development of new therapeutic targets and strategies.
